# Flexible bronchoscopy insufflated and high-flow nasal oxygen pilot trial (BUFFALO protocol pilot trial)

**DOI:** 10.1186/s40814-024-01464-w

**Published:** 2024-02-29

**Authors:** Susan Humphreys, Andreas Schibler, Tara Williams, Susan Spall, Trang Pham, Tiffany Atkins, Vikas Goyal, David Sommerfield, Aine Sommerfield, Adam Keys, Neil Hauser, Britta S von Ungern-Sternberg

**Affiliations:** 1 Department of Anaesthesia, Queensland Children’s Hospital, South Brisbane, Queensland Australia; 2 Children’s Health Research Centre, The University of Queensland, Brisbane, Australia; 3 Wesley Research Institute, Wesley Hospital, Auchenflower, Australia; 4 Critical Care Research Group, St Andrew’s War Memorial Hospital, Spring Hill, Queensland Australia; 5 Institute for Evidence-Based Healthcare, Bond University, Robina, Queensland Australia; 6 Australian Centre for Health Services Innovation, Queensland University of Technology, Brisbane, Queensland Australia; 7 Department of Respiratory and Sleep Medicine, Queensland Children’s Hospital, South Brisbane, Queensland Australia; 8 Department of Paediatrics, Gold Coast Health, Gold Coast, Queensland, Australia; 9 Department of Anaesthesia and Pain Medicine, Perth Children’s Hospital, Nedlands, Western Australia Australia; 10 Division of Emergency Medicine, Anaesthesia and Pain Medicine, Medical School, The University of Western Australia, Perth, Western Australia Australia; 11 Perioperative Medicine Team, Perioperative Care Program, Telethon Kids Institute, Nedlands, Western Australia Australia

**Keywords:** High-flow, Hypoxaemia, Anaesthesia, Paediatric, Children

## Abstract

**Background:**

Hypoxaemia occurs in approximately 30% of children during anaesthesia for flexible bronchoscopy. High-flow nasal oxygen (HFNO) can prolong safe apnoea time and be used in children with abnormal airways. During flexible bronchoscopy, there is limited evidence if HFNO confers advantages over current standard practice in avoiding hypoxaemia. The aim is to investigate feasibility of HFNO use during anaesthesia for flexible bronchoscopy to reduce frequency of rescue oxygenation and hypoxaemia.

**Methods:**

BUFFALO is a bi-centre, unmasked, randomised controlled, parallel group, protocol for a pilot trial comparing HFNO techniques to standard practice during anaesthesia. Children (*n* = 81) aged > 37 weeks to 16 years presenting for elective bronchoscopy who fulfil inclusion but not exclusion criteria will be randomised prior to the procedure to HFNO or standard care oxygenation post induction of anaesthesia. Maintenance of anaesthesia with HFNO requires total venous anaesthesia (TIVA) and with standard, either inhalational or TIVA at discretion of anaesthetist in charge of the patient. Outcomes will include the feasibility of recruitment and adherence to trial procedures, acceptability of the intervention of the protocol and completion rates of data collection methods.

**Discussion:**

Findings of this trial will determine feasibility to plan for a larger multicentre randomised clinical trial and support the feasibility of the proposed study procedures.

**Trial registration:**

BUFFALO trial was registered with Australia and New Zealand Clinical Trials Registry (TRN12621001635853) on 29 November 2021 and commenced recruitment in May 2022. https://www.anzctr.org.au/. The primary manuscript will be submitted for publication in a peer-reviewed journal.

**Supplementary Information:**

The online version contains supplementary material available at 10.1186/s40814-024-01464-w.

## Introduction

Optimal airway management during anaesthesia for paediatric flexible bronchoscopy is primarily aimed to avoid hypoxaemia as well as provide good conditions for an uninterrupted procedure [1]. Infants and children with abnormal upper and lower airways are at a higher risk of hypoxaemia during anaesthesia for flexible bronchoscopy where the airway is viewed under direct vision by the respiratory physician and then instrumented to view the lower airways [2]. This procedure requires significant collaboration and teamwork between the anaesthetist and respiratory physician as both share the same operation field, the upper airway [3]. The anaesthetist must provide adequate oxygenation and depth of anaesthesia to maintain spontaneous breathing of the child during the procedure without intubating the child’s trachea as this obstructs the view of the upper and lower airways. The onset of oxygen desaturation, as a surrogate measure of hypoxaemia in infants and children, is much faster than in adults and is known to be age-dependent [4, 5]. In small infants, apnoea can occur frequently and therefore balancing adequate depth of anaesthesia with spontaneous ventilation is often difficult in this age group. A recent survey conducted in China of more than 22,000 flexible bronchoscopies described hypoxaemia occurrence as high as 85% [6]. Our recent review of the electronic anaesthesia record from a full year, pre-COVID (2019) of all 185 elective flexible bronchoscopies in children at Queensland Children’s Hospital identified that hypoxaemia (transcutaneous oxygen saturation SpO_2_ of < 90%) occurred overall in 28% of children: when stratified by age, 32% of age under 1 year, 32% of age 1–5 years and 16% of age 5–16 years (unpublished own data). All these children received standard oxygenation via a facemask or laryngeal mask attached to the anaesthetic circuit. High-flow nasal oxygen (HFNO) therapy in children to improve oxygenation during flexible bronchoscopy and tubeless upper airway surgery is discussed as an alternative oxygenation method [7–9]. Infants and children experience periods of hypoventilation and apnoea during airway procedures; during these episodes, HFNO may bridge these children from apnoea until regained spontaneously breathing without hypoxaemia [4]. A small clinical trial in children undergoing flexible bronchoscopy conferred some advantage of HFNO over standard oxygen therapy [10].

### Knowledge gap

To date, no rigorous evaluation of HFNO during paediatric bronchoscopy has been undertaken. In this pilot study, we aim to evaluate the feasibility and efficacy of HFNO during paediatric bronchoscopy in a randomised controlled trial.

*We hypothesise* that the heated and humidified high-flow of 100% oxygen could confer advantages over standard techniques during anaesthesia of spontaneously breathing infants or children undergoing paediatric flexible bronchoscopy.

*The primary objective* of the study is to evaluate the feasibility of HFNO during paediatric anaesthesia and evaluate the efficacy of HFNO compared to standard care oxygenation techniques to prevent hypoxaemia during the procedure.

## Methods

The Standard Protocol Items: Recommendations for Interventional Trials (SPIRIT 2013) explanation and elaboration: guidance for protocols of clinical trials and the CONSORT extension to randomised pilot and feasibility trials was used to guide protocol and study design [11, 12].

### Design and settings

BUFFALO is a randomised clinical pilot trial conducted in 2 tertiary paediatric anaesthesia centres in Australia (Queensland Children’s Hospital, Brisbane and Perth Children’s Hospital, Perth).

### Participants

Children will be identified and recruited within the participating hospitals by screening of consecutive elective bronchoscopy lists. Patients meeting all inclusion criteria of ages between 37 weeks gestation to 15 years and 364 days for flexible bronchoscopy and no exclusion criteria can be enrolled (Table 1).

**Table 1:** Inclusion and exclusion criteria for BUFFALO pilot trial

**Inclusion criteria**	**Exclusion criteria**
Age > 37 weeks (corrected)–16 years (15 years + 364 days)	Choanal atresia
Elective flexible bronchoscopy	HFNO contraindication—facial trauma, cerebrospinal fluid leak
Consent by parent/guardian	Emergency procedure or out of hours
Clinicians’ approval for inclusion	

*HFNO* High-flow nasal oxygen

### Interventions

Infants and children during flexible bronchoscopy are breathing spontaneously whilst under general anaesthesia.

#### Monitoring and induction of anaesthesia

Standard monitoring for heart rate, respiratory rate and transcutaneous oxygen saturation (SpO_2_) will be applied. The additional measures of the oxygenation reserve index (ORI) and transcutaneous CO_2_, will be obtained for study purposes only. Both will remain blinded to the anaesthetist as both measures are not part of current standard practice and values. Anaesthesia will be induced as per the individual anaesthetist and IV access attained. If deemed appropriate by the anaesthetist and the proceduralist, the epiglottis, vocal cords and trachea will be visualised by direct laryngoscopy and topicalised with at least 4 mg/kg of lignocaine via a mucosal atomiser device prior to airway instrumentation. All other medication not relevant to the study outcome will be administered at the discretion of the individual anaesthetist. For both interventions, the individual recovery process from anaesthesia will be at the discretion of the attending anaesthetist. All anaesthetists involved in this study are competent in both oxygenation and anaesthesia techniques and comply with randomisation decision.

#### High-flow nasal oxygen technique

HFNO will be delivered via the Optiflow THRIVE™ system at weight specific flow rates (Table 2) delivering an inspired oxygen fraction (FiO_2_) of 1.0. Jaw thrust will be applied to ensure a patent airway until airway instrumentation begins. Anaesthesia will be maintained via total intravenous anaesthesia (TIVA) using a combination of propofol or dexmedetomidine ± an opioid at the discretion of the attending anaesthetist. A request for increased positive splinting pressure may be achieved in the HFNO group by mouth closure or application of facemask and a T-piece with HFNO discontinued during this time. A request for reduced flow rate to assess airway patency will not be a protocol deviation in this instance.

**Table 2:** High-flow rates during anaesthesia [7]

**Weight**	**High-flow rates**
0–12 kg	2 L/kg/min
13–15kg	30 L/min
16–30 kg	35 L/min
31–50 kg	40 L/min
> 50 kg	50 L/min

#### Standard care with facemask or laryngeal mask airway (LMA) technique

Oxygen insufflation will be at a flow rate of up to 6 L/min via anaesthesia facemask with Bodei connector or a second generation laryngeal mask airway (LMA). Anaesthesia will be maintained at the discretion of the anaesthetist, via inhalational anaesthesia with sevoflurane (facemask or LMA only), intravenous agents (propofol, dexmedetomidine and/or short-acting opioid), or a combination of both. Intravenous agents administered will be as per choice of the attending anaesthetist. A request for increased positive splinting pressure may be achieved in the standard care group by increasing PEEP at the adjustable pressure-limiting (APL) valve of the anaesthesia machine or occlusion of the T-piece, depending on the set-up used by the clinical team.

#### Management of hypoxaemic events requiring rescue oxygenation

A rescue oxygenation attempt occurs when the patient desaturates below a clinically accepted safe level. This rescue attempt is usually discussed and coordinated between the anaesthetist and the proceduralist. Normally, significant hypoxaemia for anaesthesia is defined as an oxygen saturation of < 90% [13–15]. However, dependent of the patient’s physiology, age and starting saturation levels prior to the procedure, the anaesthetist can accept transiently lower oxygen levels if required to allow an uninterrupted procedure. Similarly, the procedure can contribute to hypoxaemia and acceptance of this is again at the discretion of the anaesthetist and operator.

#### Rescue oxygenation

Rescue oxygenation is defined as interruption of the bronchoscopy due to hypoxaemia and the anaesthetist attempts to improve oxygenation of the child using positive pressure assisted ventilation. This study will not define at which oxygenation level the patient is rescued as it is more relevant having this defined by the individual anaesthetist (independent of the study team) using clinical discretion.

#### Concomitant investigations

For combined procedures such as tubeless upper airway procedures, imaging or tonsillectomy, this study will include the anaesthetic period of only the flexible bronchoscopy airway surgery procedure.

### Study aims, outcome measures and definitions

The primary objective of this pilot study is to assess the feasibility of conducting a larger-scale randomised controlled trial (RCT). In the future, this fully powered RCT will assess the effect of HFNO during bronchoscopy in children compared to standard practice. The current feasibility trial will identify any factors that may detract from our ability to achieve this aim in the full trial.

*Feasibility outcomes* will focus on the monthly recruitment rate, the randomised to screened patient ratio and protocol adherence.

#### Proposed efficacy outcomes

These outcomes are recorded as part of the data collection methods assessed in this feasibility study and are included in the protocol for descriptive purposes recognising that the pilot phase will not be adequately powered to detect a difference in these outcomes, and they will not be analysed as part of the feasibility assessment.

The proposed primary efficacy outcomes are as follows: (1) the proportion of children who experience a hypoxaemic event defined as a fall in peripheral oxygen saturation (SpO_2_) < 90% or > 10% of the baseline SpO_2_ for children with cyanotic heart disease and the severity of hypoxaemic defined as lowest SpO_2_ and (2) the proportion of children requiring interruption of the bronchoscopy for airway management due to hypoxaemic event. The investigators’ view is that a hypoxaemic event that requires rescue intervention irrespective of the cause is the true and important outcome measure for this study.

The proposed secondary efficacy outcomes are as follows: (1) total length of time patient experiences hypoxaemia [seconds] during hypoxaemic event and the area under the curve of the SpO_2_ signal (below (90% or >10% fall from baseline SpO_2_); (2) minor adverse events: occurrence of epistaxis, laryngospasm, bronchospasm, coughing at any time during procedure; (3) major adverse events: occurrence of hypotension requiring treatment, bradycardia requiring treatment, cardiac arrest with or without return of spontaneous circulation at any time during procedure; (4) requirement for unexpected PICU or high dependency unit (HDU) admission; (5) requirement for unanticipated post-bronchoscopy mechanical ventilation or any other form of non-invasive ventilation including nasal high-flow; (6) length of PICU or HDU stay if admitted post procedure; (7) length of hospital stay.

### Study procedures

All theatre lists including upper airway surgeries will be screened for eligible patients. A screening log will record patient information including name, unique reference number, eligibility, enrolment and treatment allocation. Baseline and outcome parameters will be collected for all eligible and consented children by a dedicated research team member. Patient demographics will include age, weight, American Society of Anesthesiologist (ASA) classification, comorbidities including recent lung pathologies and preoperative oxygen or ventilatory support, diagnosis and indication for procedure, and bronchoalveolar lavage. Clinical parameters throughout the entire procedure from induction to end of anaesthesia will include the following: transcutaneous oxygen saturation (SpO_2_), heart rate (HR), respiratory rate (RR), non-invasive blood pressure (NIBP), end-tidal CO_2_ (ETCO_2_) (for HFNO, pre-application of cannulae and post procedure), length of procedure, fresh gas flow rate, oxygen reserve index (ORI) via Masimo SET® (Masimo, Irvine, CA, USA), and transcutaneous CO_2_ and O_2_ saturations which will be acquired continuously via TCM Flex Monitor (Radiometer, Copenhagen, Denmark). All signals will be continuously recorded using the ICM+ software (Cambridge, UK). Cormack and Lehane classification on direct laryngoscopy will be noted and length of stay in post anaesthetic care unit.

### Participant recruitment and randomisation

**Fig. 1:**
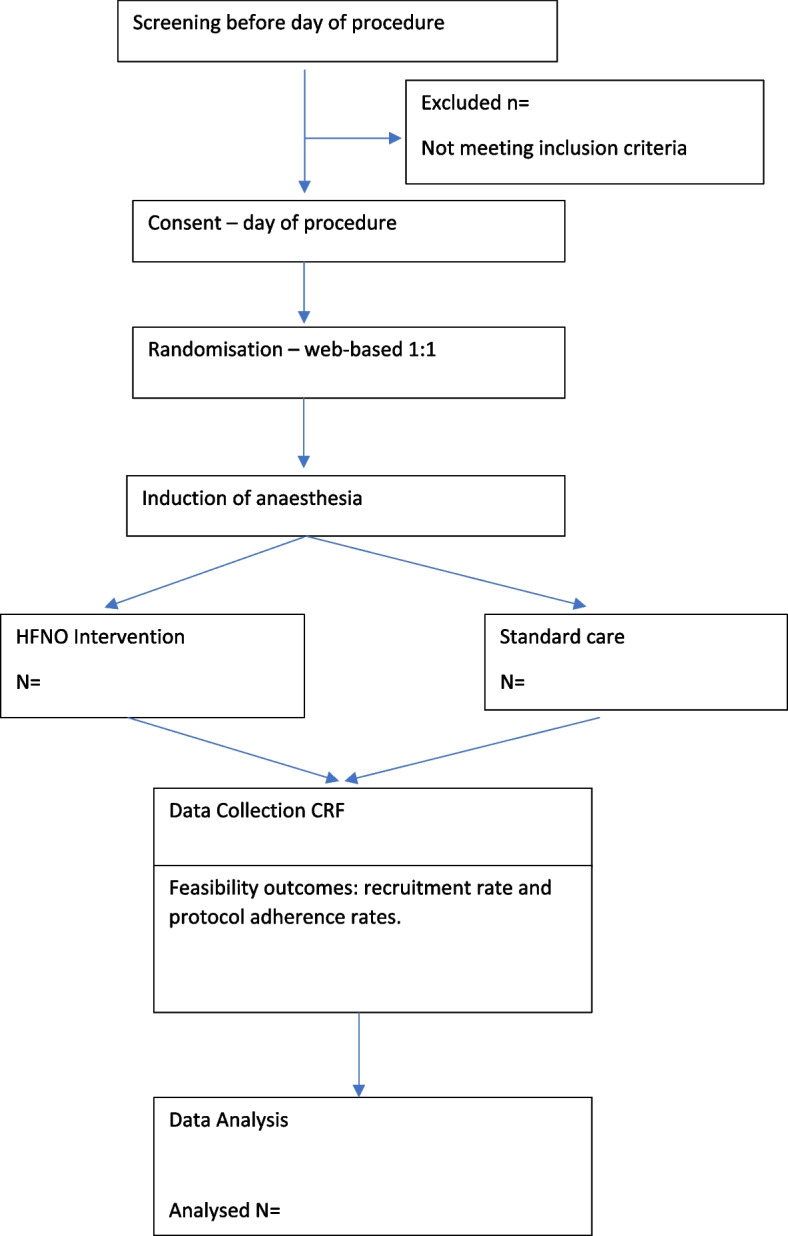
Timeline of study procedures

Eligible participants will be screened and recruited by a trained research team member on the day of the procedure, prior to the procedure (Fig. 1).

#### Randomisation and blinding

Patients will be allocated to a treatment arm using a centralised web-based randomisation tool with an allocation of 1:1 either to HFNO group or standard care group. Randomisation will be stratified by age (< 1 year, 1 < 5 year and 5–16 years of age) and site with variable block sizes within each stratum. Patients will be allocated to a study arm immediately after consent has been achieved to allow enough time to prepare the relevant equipment and drugs for anaesthesia. It is not possible to blind anaesthetists or outcome assessors to treatment allocation due to the nature of the intervention, but the data will remain blinded to the analysis team until recruitment and data cleaning has been completed.

#### Post consent and randomisation exclusion

Patients who fulfil the inclusion criteria and no exclusion criteria at the time of screening and who are consented and randomised but the case is cancelled or do not have a flexible bronchoscopy during their theatre time will be excluded from the primary analysis dataset as a post consent and post randomisation exclusion.

## Sample size, data management and statistical analysis plan

As this is a feasibility study, the sample size of this pilot trial is based on the recruitment rate and on the recruitment rate to retention ratio within a 1-year recruitment to allow seasonal variations.

### Feasibility outcomes

We aim to achieve a 30% recruitment rate of eligible patients, 90% protocol adherence and 100% of follow-up assessment based on being an established research centre with previously completed pilot studies achieving these targets.

The purpose of pilot studies is not to test hypotheses, and inferential statistics are therefore not required. Descriptive statistics will predominantly be used to summarise participant characteristics and outcome measures. Whilst it is acknowledged that the smaller sample sizes associated with pilot studies can provide unstable effect size estimates, we intend to carry out some exploratory analyses alongside descriptive statistics for efficacy outcomes. We will note—but not statistically account for—observed differences between groups at baseline. Measures of variance on this outcome variable will be used to inform the design of a definitive RCT.

#### Recruitment rate

The recruitment rate (expressed as the proportion of recruited patients over total eligible patients) will be assessed after 81 consecutively recruited patients.

#### Protocol deviations

Protocol deviations will be recorded in the patient record (source document) and on the CRF. Protocol deviations will be assessed for significance by the investigators. Those deviations deemed to have a potential impact on the integrity of the study results, patient safety or the ethical acceptability of the trial will be reported to the HREC in a timely manner. Where deviations to the protocol identify issues for protocol review, the protocol will be amended. A protocol deviation will be defined as ‘the incorrect intervention was used for a portion of study enrolment’.

### Sample size

The sample size of 81 participants will ensure that the width of the 95% confidence intervals for the 3 feasibility outcomes are between (plus or minus) 5% and 10%.

#### Sample size for definitive trial

Our own data obtained at the Queensland Children’s Hospital, Brisbane, showed that hypoxaemia occurred overall in 28% of children: when stratified by age, 32% of age under 1 year, 32% of age 1–5 years and 16% of age 5–16 years. Assuming 90% power and a type 1 error rate of 0.05 with a 15% attrition rate included requires, based on current data, a total sample size of 416 for the full trial to reduce the incidence of hypoxaemia from 28 to 14%. However, the sample size will be adjusted after completion of the pilot trial using the pilot data on hypoxaemic events.

#### Statistical analysis plan

Descriptive statistics will be used to report on the baseline characteristics of the total study cohort and each treatment group as well as by site. As this is a pilot trial, formal statistical comparisons will not be undertaken. All outcomes will be presented as estimate along with 95% confidence interval (CI); feasibility outcomes will be presented only for the total cohort, whilst safety and efficacy outcomes will be presented for the total cohort as well as per treatment group. For the three feasibility outcomes, the proportion in each intervention arm will be compared using a Fisher exact test. Analyses will be undertaken using intention-to-treat; however, the number and type of protocol deviations will be reported to assist in feasibility assessment.

To inform the future trial, where appropriate, statistical analysis will be performed by intention-to-treat.

### Data management

A data dictionary will be prepared and maintained for the duration of the study. Data will be entered into the electronic data platform REDCap™ (Research Electronic Data Capture, Vanderbilt http://project-redcap.org/) version 7 hosted by the University of Queensland. Hard copy and electronic site data will be stored securely on password protected computers and paper CRFs in locked filing cabinets in a secure location at each hospital site. In keeping with the Queensland Health Retention and Disposal Schedule 2DAN546 v3 (Clinical research records for minors), the information will be retained for 15 years. A permanent record of the patient’s participation in the trial will also be located in the patient record. This also fulfils the Australian Code for the Responsible Conduct of Research requirements for retention of research data. Missing data will be considered in analyses and reported in the results of the trial. On-going surveillance and adherence to the study protocol (intervention fidelity) will be monitored by the principal investigator (PI) and clinical research assistant (CRA) during weekly audits Fig. 1.

### Data safety and monitoring

An independent data and safety and monitoring board (DSMB), comprising experts in clinical trials and anaesthetic care, has been established before patient enrolment to review the trial protocol. The DSMB will meet after the first 20 patients and then after 61 patients and at the completion of recruitment. The DSMB will be forwarded a copy of all serious adverse events (SAE) reports as soon as they become available. The DSMB will review all SAE reports that they receive and report back to the CIs of the study if any further action is required. SAEs are defined in accordance with The Australian Clinical Trial Handbook and include any event that is fatal, life-threatening, permanently disabling, incapacitation or prolongs a hospital stay [16]. All SAEs will be reported to the site Ethics committee within 48 h.

### Patient and public involvement

Both PCH and QCH were involved in a consumer-led ACORN study (Anaesthetic Consumer Research Network), which set research priorities for anaesthesia and pain management in children. The ACORN study involved more than 850 Australian consumers, and the number one priority for consumers was identified as safer anaesthesia for children [17]. Parents placed high importance on research to minimise complications, particularly respiratory complications or hypoxaemia which are the most common complications in paediatric anaesthesia practice. Airway procedures, due to the associated high risk for complications and adverse outcomes, have been highlighted as an area of particular concern and need for research from the ACORN clinician surveys. Furthermore, since study conception, this study has been discussed with the Consumer Research Reference Panel of the Anaesthesia Research Team at PCH. The panel includes consumer representatives from various socioeconomic backgrounds as well as those from culturally and linguistically diverse backgrounds and First Nation members.

### Patient consent for publication

Not required.

### Ethics approval

Ethics approval was obtained for the BUFFALO Trial from Children’s Health Queensland HREC/21/QCHQ/76582. The BUFFALO Trial is registered with the Australian and New Zealand Clinical Trial Registry (ACTRN12621001635853).

### Dissemination

All dissemination will involve aggregate data only and be undertaken using the CONSORT 2010 statement: updated guidelines for reporting parallel group randomised trials [18] and the TIDIER checklist [19].

## Trial status

Recruitment of patients to BUFFALO commenced in 3 May 2022. We expect to complete the recruitment within 18 months from date of commencement.

## Trial endorsement

This trial has received Australian and New Zealand College of Anaesthetist Clinical Trial Network (ANZCA CTN) endorsement.

## Discussion

We are currently undertaking a bi-centre, pilot RCT in two hospitals to address the question of feasibility and the efficacy of HFNO as an oxygenation method during anaesthesia for children undergoing flexible bronchoscopy by using clinical outcomes that are clinically important and also consumer informed for safety of the anaesthesia. Findings of this trial will determine feasibility to plan for a larger multicentre randomised clinical trial and support the feasibility of the proposed study procedures. Our progression criteria will require all three of the following: achieving a 30% recruitment rate of eligible patients, 90% protocol adherence and 100% of follow-up assessment. High-quality evidence derived from a full efficacy trial will enable the successful translation and adoption of this new technique into paediatric anaesthesia practice and will answer important aspects of clinical cost effectiveness.

### Supplementary Information

**Additional file 1: Appendices.** 

## Abbreviations

ACORN: Anaesthetic Consumer Research Network
APL: Adjustable pressure limiting
ASA: American Society of Anesthesiologists Classification
BUFFALO: Bronchoscopy insufflated and high-flow nasal oxygen
CI: Confidence interval
CRA: Clinical research assistant
DSMB: Data and safety monitoring board
EtCO_2_: End-tidal carbon dioxide
FiO2: Fraction of inspired oxygen
HDU: High dependency unit
HFNO: High-flow nasal oxygen
LMA: Laryngeal mask airway
NIBP: Non-invasive blood pressure
ORI: Oxygen reserve index
PEEP: Positive end-expiratory pressure
PICU: Paediatric intensive care unit
RCT: Randomised controlled trial
REDCap: Research Electronic Data Capture
RR: Respiratory rate
SAE: Serious adverse events
SpO_2_: Transcutaneous oxygen saturation
TCO_2_: Transcutaneous carbon dioxide
TIVA: Total intravenous anaesthesia

## References

[1] Bass JL, Corwin M, Gozal D (2004). The effect of chronic or intermittent hypoxia on cognition in childhood: a review of the evidence. Pediatrics.

[2] Mausser G, Friedrich G, Schwarz G (2007). Airway management and anesthesia in neonates, infants and children during endolaryngotracheal surgery. Paediatr Anaesth.

[3] Londino AV, Jagannathan N (2019). Anesthesia in diagnostic and therapeutic pediatric bronchoscopy. Otolaryngol Clin North Am.

[4] Humphreys S, Lee-Archer P, Reyne G, Long D, Williams T, Schibler A (2017). Transnasal humidified rapid-insufflation ventilatory exchange (THRIVE) in children: a randomized controlled trial. Br J Anaesth.

[5] Patel R, Lenczyk M, Hannallah RS, McGill WA (1994). Age and the onset of desaturation in apnoeic children. Can J Anaesth.

[6] Lin J, Tao X, Xia W (2021). A multicenter survey of pediatric flexible bronchoscopy in western China. Transl Pediatr.

[7] Humphreys S, Rosen D, Housden T, Taylor J, Schibler A (2017). Nasal high-flow oxygen delivery in children with abnormal airways. Paediatr Anaesth.

[8] Riva T, Theiler L, Jaquet Y, Giger R, Nisa L (2018). Early experience with high-flow nasal oxygen therapy (HFNOT) in pediatric endoscopic airway surgery. Int J Pediatr Otorhinolaryngol.

[9] Baker PA, Rankin L (2019). Successful application of optiflow THRIVE to restore oxygenation and facilitate retrieval of an aspirated nut in a severely hypoxic child: a case report. A&A practice.

[10] Sharluyan A, Osona B, Frontera G (2021). High flow nasal cannula versus standard low flow nasal oxygen during flexible bronchoscopy in children: a randomized controlled trial. Pediatr Pulmonol.

[11] Chan AW, Tetzlaff JM, Gotzsche PC (2013). SPIRIT 2013 explanation and elaboration: guidance for protocols of clinical trials. BMJ.

[12] Eldridge SM, Chan CL, Campbell MJ (2016). CONSORT 2010 statement: extension to randomised pilot and feasibility trials. Pilot Feasibility Stud.

[13] Motoyama EK, Glazener CH (1986). Hypoxemia after general anesthesia in children. Anesth Analg.

[14] Pullerits J, Burrows FA, Roy WL (1987). Arterial desaturation in healthy children during transfer to the recovery room. Can J Anaesth = Journal canadien d'anesthesie.

[15] Vijayakumar HR, Metriyakool K, Jewell MR (1987). Effects of 100% oxygen and a mixture of oxygen and air on oxygen saturation in the immediate postoperative period in children. Anesth Analg.

[16] Therapeutic Goods Administration. The Australian clinical trial handbook. Australian Government. 2006.

[17] Sommerfield A, Sommerfield D, Bell E (2023). Consumer research priorities for pediatric anesthesia and perioperative medicine. Paediatr Anaesth.

[18] Schulz KF, Altman DG, Moher D (2010). CONSORT 2010 statement: updated guidelines for reporting parallel group randomised trials. BMJ.

[19] Hoffmann TC, Glasziou PP, Boutron I (2014). Better reporting of interventions: template for intervention description and replication (TIDieR) checklist and guide. BMJ.

