# Supplementary material for: Flexible bronchoscopy insufflated and high-flow nasal oxygen pilot trial (BUFFALO protocol pilot trial)
Source: Pilot Feasibility Stud. 2024 Feb 29;10:45. doi: 10.1186/s40814-024-01464-w (PMC10902996; doi:10.1186/s40814-024-01464-w)
Supplement: Supplementary file 1 — Additional file 1: Appendices. [file 40814_2024_1464_MOESM1_ESM.docx]

**APPENDICES**

**CHILD/ADOLESCENT INFORMATION SHEET**


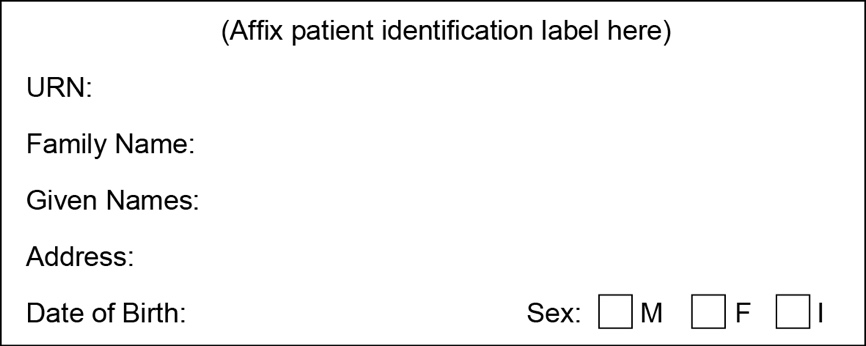


*(Local Site Logo)*

**Project Title Flexible Bronchoscopy Insufflated and High-Flow Nasal Oxygen Pilot Trial (BUFFALO)**

**HREC Number**

**Investigator A/Prof Susan Humphreys [*Local site investigator(s)*],**

**Version: Version 1: 01.10.2021**

#### Thank you for taking the time to read this **Information Sheet**. We would like to ask you to participate in a **research project** that is explained below.

**It is ok to say no**

**Introduction**

We would like to invite you to participate in this study. You are having a Flexible Bronchoscopy to look in your lungs with a special camera. While we are looking into lungs, we will give you oxygen to breathe. We have different ways to give you the oxygen and in this project we are investigating a new method for this procedure.

This is an exciting project and your participation could lead to changes in how we do deliver oxygen for these procedures in the future.

**Description of Experiment – methods and demands**

While you are asleep, we will give you oxygen to breathe. This will happen while they are looking into your lungs with the special camera. During this time, we will have special monitors on you to keep you safe during the procedure.

At the end of the Flexible Bronchoscopy we will wake you up and give you more oxygen through a clear mask on your face. This will be the end of our project. You will then wake up and recover with your parents/caregiver.

**Risk Discomfort**

When you are having your Flexible Bronchoscopy you will be asleep. We will give you oxygen either through your nose or through a small device we put in your mouth. You may wake up with a dry mouth or a sore throat. This should not last for long and we can help you when waking up with either of these problems.

**Benefits**

Through this project we are trying to determine which is the best way to give children oxygen when they are having these types of procedures. Your involvement will help us to investigate this.

**Withdrawing from the Study**

Your decision whether or not to participate will not prejudice your future relations with the Children’s Health services. If you decide to participate, you are free to withdraw your consent and to discontinue participation at any time. The decision to withdraw from the study will not affect your routine medical treatment or your relationship with the people treating you

You can talk to your parents/caregivers or the Doctors or Nurses looking after you today about the project and ask any questions that you might have.
